# Supplementary material for: Fossil eggshell cuticle elucidates dinosaur nesting ecology
Source: PeerJ. 2018 Jul 6;6:e5144. doi: 10.7717/peerj.5144 (PMC6037156; doi:10.7717/peerj.5144)
Supplement: Supplemental Information 1 — Detailed descriptions of the applied methodology and further material information. [file peerj-06-5144-s001.docx]

**Supplemental Information**

1. **Description of eggshell specimens**

Elongatoolithidae Zhao, 1975

*Macroolithus* Zhao, 1975

*Macroolithus yaotunensis* Zhao, 1975

(Laid by the oviraptorid *Heyuannia huangi*)


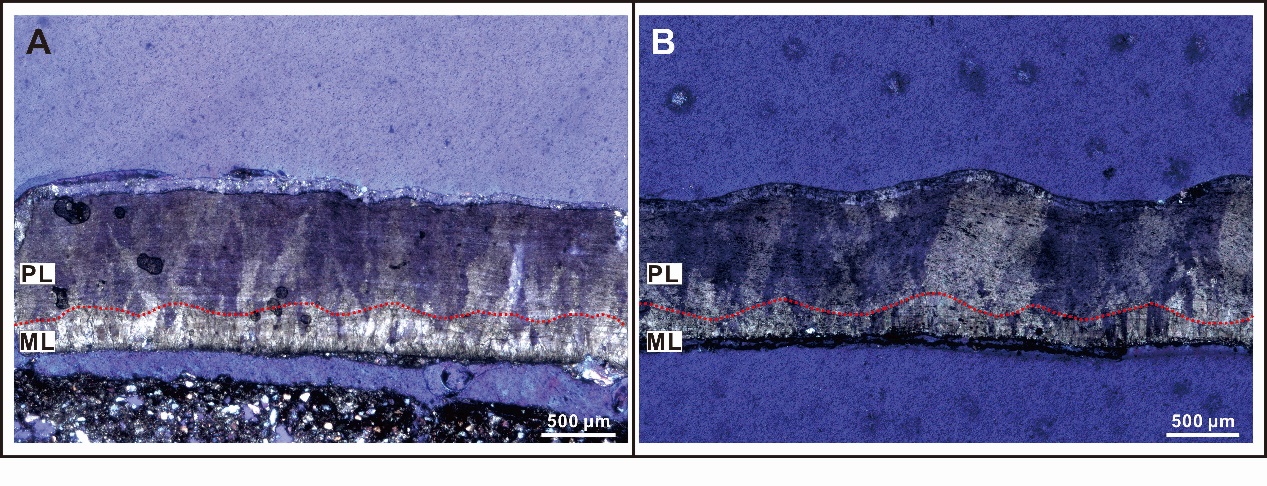


**Figure S1** Photographs of the *Macroolithus yaotunensis* sample from (A) the Liguanqiao Basin in Henan and (B) Nanxiong Basin in Guangdong Provinces, China under the light polarized microscope. The red dashed line marks the undulating boundary between the prismatic layer and mammillary layer, which characterizes the oospecies *Macroolithus huangi*. ML, mammillary layer; PL, prismatic layer.

1. **Raman band assignment**

| Wavenumbers | Assignment | References |
| --- | --- | --- |
| 957-962 | ν_1_PO4, P–O | Crane et al., 2006 |
| 967 | ν_1_ symmetric $\mathrm{PO}_{4}^{3-}$ stretching | Walters et al., 1990; Gergely et al. 2010; Frost et al., 2014; Igic et al., 2015 |
| 970 | ν_1_PO4, P–O | Crane et al., 2006; Igic et al., 2015 |
| 980 | $\mathrm{PO}_{4}^{3-}$ or $\mathrm{HPO}_{4}^{2-}$ | Sauer et al., 1994; Crane et al., 2006; Igic et al., 2015 |
| 1087 | ν_1_ symmetric CO_3_ stretching | Gunasekaran, Anbalagan & Pandi, 2006 |

**Reference**

Gergely G, Wéber F, Lukács I, Tóth AL, Horváth ZE, Mihály J, Balázsi C. 2010. Preparation and characterization of hydroxyapatite from eggshell. Ceramics International 36:803-806. DOI: 10.1016/j.ceramint.2009.09.020

Gunasekaran S, Anbalagan G, Pandi S. 2006. Raman and infrared spectra of carbonates of calcite structure. *Journal of Raman Spectroscopy* 37(9):892-899. DOI:10.1002/jrs.1518

Igic B, Fecheyr-Lippens D, Xiao M, Chan A, Hanley D, Brennan PRL, Grim T, Waterhouse GIN, Hauber ME, Shawkey MD. 2015. A nanostructural basis for gloss of avian eggshells. *Journal of The Royal Society Interface* 12:20141210. DOI: 10.1098/rsif.2014.1210

Walters MA, Leung YC, Blumenthal NC, Konsker KA, LeGeros RZ. 1990. A Raman and infrared spectroscopic investigation of biological hydroxyapatite. *Journal of Inorganic Biochemistry* 39:193-200. DOI: 10.1016/0162-0134(90)84002-7

Zhao, Z. 1975. Microstructures of the dinosaurian eggshells of Nanxiong, Guangdong, and the problems in egg classification. *Vertebrata Palasiatica* 13(2):105-117.
